# High Pressure Extraction of Antioxidants from *Solanum stenotomun* Peel

**DOI:** 10.3390/molecules18033137

**Published:** 2013-03-08

**Authors:** Lourdes Casas Cardoso, Casimiro Mantell Serrano, Edwin Torrez Quintero, Clara Pereyra López, Ruder Medrano Antezana, Enrique J. Martínez de la Ossa

**Affiliations:** 1 Department of Chemical Engineering and Food Technology, Faculty of Science, University of Cadiz, Campus de Excelencia International Agroalimentario ceiA3, Box 40, 11510 Puerto Real, Cadiz, Spain; E-Mails: casimiro.mantell@uca.es (C.M.S.); clara.pereyra@uca.es (C.P.L.); enrique.martínezdelaossa@uca.es (E.J.M.O.); 2 Food and Natural Products Center, Faculty of Sciences and Technology, University of San Simon, Cochabamba 4737, Bolivia; E-Mails: torresedwin@yahoo.com (E.T.Q.); rmedrano@hotmail.com (R.M.A.)

**Keywords:** high pressure extraction, potato peels, anthocyanins, antioxidants, chromatography, selectivity

## Abstract

In the work described here, two techniques for the recovery of anthocyanins from potato peel were studied and compared. One of the techniques employed was supercritical fluid extraction (SFE) with pure CO_2_ or with CO_2_ and ethanol as cosolvent and the other technique was pressurized liquid extraction (PLE), where the solvent used was ethanol in water acidified to pH 2.6. The effects of pressure and temperature were studied and the anthocyanin contents obtained were statistically analyzed. In SFE the use of low pressure (100 bar) and high temperature (65 °C) was desirable for the anthocyanin extraction. With PLE the anthocyanin contents are increased considerably, and the best yields were obtained at 100 bar and 80 °C. This result is in correspondence with antioxidant activity index values (1.66) obtained in a DPPH antioxidant activity assay. In the extracts obtained with PLE the phenolic compounds were also determined, but the main compounds presented in the extract are anthocyanins.

## 1. Introduction

Free radical biology is a burgeoning discipline that has been investigated more widely in life science in recent years. This area mainly concerns the formation and scavenging of free radicals, as well as the damage caused by free radicals in biological systems. It is now well established that a series of oxygen-centred free radicals and other reactive oxygen species contribute to the pathology of numerous disorders, including atherogenesis, neurodegeneration, chronic inflammation, cancer and physiological senescence [1], therefore, antioxidants are considered to be important nutraceuticals on account of their many health benefits and they are widely used in the food industry as potential inhibitors of lipid peroxidation [2]. However, it has been demonstrated that synthetic antioxidants can accumulate in the body and this can result in liver damage and carcinogenesis. These problems are not seen when natural antioxidants, which are extracted from herbs and spices and have high antioxidant activity, are used in food applications. These extracts are considered safe, potentially nutritional and to have therapeutic effects.

Flavonoids are a group of polyphenolic compounds that are present in many natural products and have been studied extensively due to their high antioxidant, anti-microbial and antiproliferative activity [3,4]. Apart from their health benefits and nutraceutical value, anthocyanins are also used as food colorants since their color is pH dependent. Fundamental studies have indicated that at different pH levels, anthocyanins undergo structural transformations that are responsible for their color changes in solution.

Potato peel is a waste product generated from potato processing and disposal of this material is a problem. Many varieties of potato contain large amounts of antioxidant pigments in the tuber and, for this reason, potato peel is a good source of functional ingredients. The variety ‘Pinta-Boca’ is cultivated in different part of South America and it has a very high anthocyanin content in the peel.

In recent years, numerous methodologies for the extraction of compounds of relatively high polarity have been developed that employ different extraction methods. In the present work we analyze and compare the viability of two anthocyanin extraction methods: supercritical fluid extraction (SFE) and pressurized liquid extraction (PLE).

SFE was introduced as an environmentally benign and efficient extraction technique for solid materials [5]. In most of these studies carbon dioxide is used as the solvent because of its relatively low critical temperature, non-toxicity, non-flammability, good solvent power, ease of removal from the product and low cost. However, quantitative extraction of polar analytes, such as anthocyanins, typically requires the addition of an organic modifier [6].

PLE is based on the use of conventional solvents at controlled temperatures and pressures and this approach for the extraction of valuable compounds from natural sources is well established. PLE requires less solvent and the extraction is complete in a shorter period of time in comparison to traditional organic solvent extraction. Subcritical water at temperatures between 100 and 180 °C has been identified as optimal to recover the maximum amount of anthocyanins from berry substrates, grape skins [7] and blackcurrants [8]. Several authors [9] found that subcritical water extraction at 160 to 180 °C, 6 MPa and 60 min is a good substitute for organic solvents such as methanol and ethanol to obtain phenolic compounds from potato peel. However, at these temperatures there is a greater possibility of thermal degradation of the flavonoid compounds during the extraction from grape pomace.

Studies performed on black carrots using water acidified with citric and lactic acids at temperatures between 50 and 100 °C and 50 bar pressure yielded the maximum amount of anthocyanins, particularly when lactic acid was used as the acidulant [10]. These studies showed that greater thermal degradation of the anthocyanin extracts occurred at 100 °C, along with higher concomitant browning when sulfuric acid was used as an acidulant. In this study, the extractions at high temperatures were performed for only 10 min, while the sulfuric acid extractions were performed at room temperature over a period of 12 h. It should be noted that in the aforementioned studies with mineral and/or organic acids, the pH of the extraction medium was not reported.

Recently, studies performed by Monrad *et al.* [11] indicated that 50%–70% v/v ethanol in water and temperatures between 80 and 120 °C was optimal for the recovery of anthocyanins and procyanidins from grape pomace.

Several other methods have been applied for extraction of antioxidants from plant matrices, one such novel process being microwave assisted extraction (MAE). MAE presents different advantages regarding to conventional methods: it is faster, selective and lower solvent consumption [12,13]. MAE has been used to extract phenolic antioxidant from potato peels; Singh *et al.* [14] used a response surface method for to optimize the MAE parameters such as extraction time, solvent (methanol) concentration and microwave power level for extraction of antioxidants from potato peels. They were able to extract higher levels of phenolic from dried potato peel than the values reported by number of previous studies. 

A MAE method coupled with the orthogonal array design was investigated for efficient extraction of the phenolic compounds in potato downstream wastes [15]. Four parameters were examined for the MAE of the total phenolic content and optimized at 60 % ethanol, 80 °C, 2 min, solid-to-solvent ratio 1:40 (g/mL). The MAE was proven more efficient than the conventional solvent extraction by refluxing.

The advantage of PLE and SFE over MAE is the applicability at different scales. PLE and SFE can be applied to systems on various scales, from the laboratory scale (a few grams) to the pilot plant scale (several hundred grams of sample), through to the industrial scale (tons of raw materials). 

It is worth to mention that SFE and PLE have been widely used for extraction process development, that is, to extract bioactive compounds from different matrices. Even though these processes usually offer clear advantages over traditional ones, the main drawback for industrial scale use is the lack of realistic economic studies. In this sense, some articles have been lately published dealing with the assessment of the industrial economic feasibility of some developed process, such as essential oil extraction from rosemary, fennel and anise [16] and brewery spent grain management [17]. Moreover, industrial applications of SFE have experienced a strong development since the early 1990s in terms of patents [18]. Therefore, SFE can be regarded as a possible tool not only from a laboratory point of view but also for the natural products and food industries.

In the work described here, a comparison of two high-pressure extraction methods for the recovery of antioxidant compounds (anthocyanins) from *Solanum stenotomun* has been analyzed. The methods analyzed were Supercritical Fluid Extraction (SFE) studying the effect of ethanol as cosolvent, and Pressurized Liquid Extraction (PLE) using ethanol as solvent. The effects of temperature and pressure on the total extraction yield, anthocyanin and phenolic content were studied. The antioxidant capacity of the extract obtained at the best conditions was measured and compared with data obtained from the literature.

## 2. Results and Discussion

### 2.1. Supercritical Fluid Extraction (SFE)

**Figure 1:**
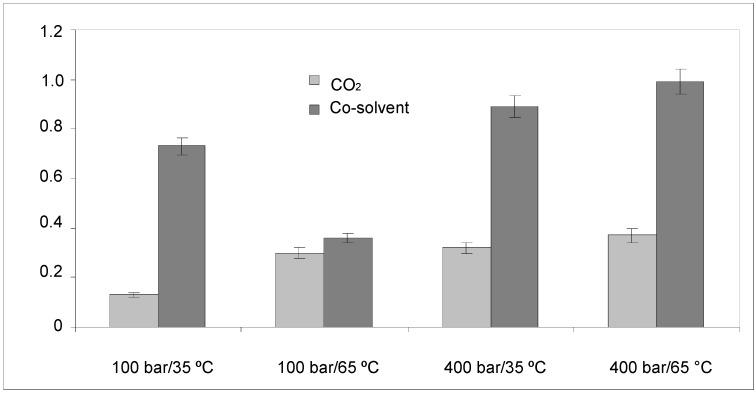
Extraction yield (%) of extracts obtained by SFE at different pressures and temperatures.

The extraction yields (%) obtained with pure CO_2_ and CO_2_ + 5% (v/v) ethanol as cosolvent are represented in Figure 1. The maximum extraction yields were obtained on addition of 5% (v/v) ethanol cosolvent to the CO_2_. This finding is attributed to the modification of the properties of the carbon dioxide—a change that enables this fluid to extract the lipophilic and hydrophilic compounds.

CO_2_ is not very suitable for the extraction of polar analytes and this problem is often tackled by the use of organic modifiers. Depending on the type of sample matrix and the affinity of the analyte for the matrix, the modifier may influence the extraction in three different ways: (1) by increasing the solubility of the analyte in the supercritical fluid as a result analyte—modifier interactions in the fluid phase; (2) facilitating analyte desorption—the molecules of polar modifiers are able to interact with the matrix and compete efficiently with the analyte for the active sites in the matrix; (3) distorting the matrix—analyte diffusion process and favor penetration of the supercritical fluid into the matrix when the modifier swells the matrix. 

The fluid pressure is an essential parameter in SFE, since fluid density is directly related to pressure. It can be observed from Figure 1 that at constant temperature, an increase in the pressure led to an increase in the extraction yield. The density of carbon dioxide is higher at higher pressure and, consequently, the solvating power of the supercritical fluid also increases. 

Temperature also has a significant effect on the recovery of components in SFE. According to the rules of kinetics, if the other variables are constant, an increase in temperature is related to a more intensive thermal motion of solutes in the active sites of the matrix. This situation is beneficial for the solutes in overcoming the adsorbing energy forces on the matrix and the solutes are desorbed more efficiently from the active sites by CO_2_ at higher temperature. From a thermodynamic point of view, the saturated vapor pressure increases with a corresponding increase in the temperature, thus enabling the solutes to dissolve in the supercritical fluid more easily. Moreover, the density of CO_2_ decreases with an increase in temperature, thus decreasing the solvency of CO_2_. These three effects compete with one another during the extraction process. The effect of temperature on the extraction yield is shown in Figure 1.

On using CO_2_ at 100 bar, an increase in the temperature from 35 °C to 65 °C led to an increase in the extraction yield; however, with CO_2_ + ethanol an increase in the temperature led to a decrease in the extraction yield. This behavior is attributed to compensation between factors on increasing the temperature. At higher pressure (400 bar), an increase in the temperature led to a slight increase in the extraction yields. 

#### 2.1.1. Anthocyanin Contents

**Figure 2:**
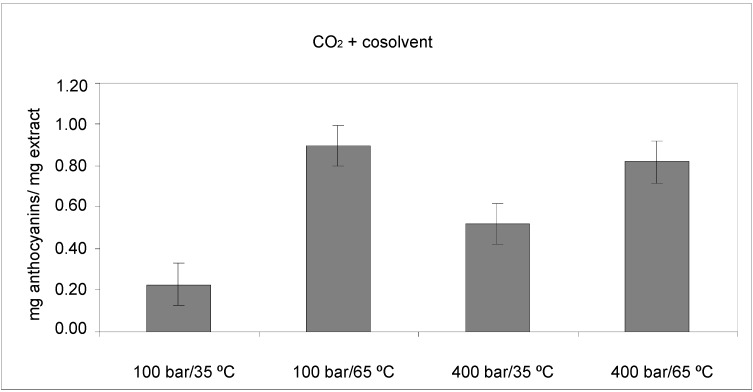
Anthocyanin concentration of extracts obtained by SFE at different pressures and temperatures.

The anthocyanin concentration (mg anthocyanins/mg dry extract) in the extracts obtained with CO_2_ + ethanol as cosolvent are shown in Figure 2. Anthocyanins were not detected by HPLC in the extracts obtained with pure CO_2_. Pressure and temperature appeared to have a marked influence on anthocyanin contents in the extracts. The use of low pressure (100 bar) and high temperature (65 °C) was desirable for the extraction of anthocyanins. These results are consistent with those reported in the bibliography by other authors for the extraction of anthocyanins from different raw materials [19].

The anthocyanin contents obtained by SFE were statistically analyzed. Regression analysis was performed on the experimental data and the coefficients of the model were evaluated for significance. It observed by ANOVA (Table 1), that pressure, temperature and the crossed interaction pressure-temperature all significantly influenced anthocyanin content (*p* ≤ 0.05).

**Table 1:** ANOVA for the effect of pressure (P) and temperature (T) on anthocyanin contents.

Source	SS	DF	MS	F-Value	*p*-value
P	0.0153125	1	0.0153125	23.71	0.0165
T	0.446512	1	0.446512	691.37	0.0001
PT	0.0861125	1	0.0861125	133.34	0.0014
Blok	0.0003125	1	0.0003125	0.48	0.5367
Total error	0.0019375	3	0.000645833		
Total (corr.)	0.550188	7			

**Figure 3:**
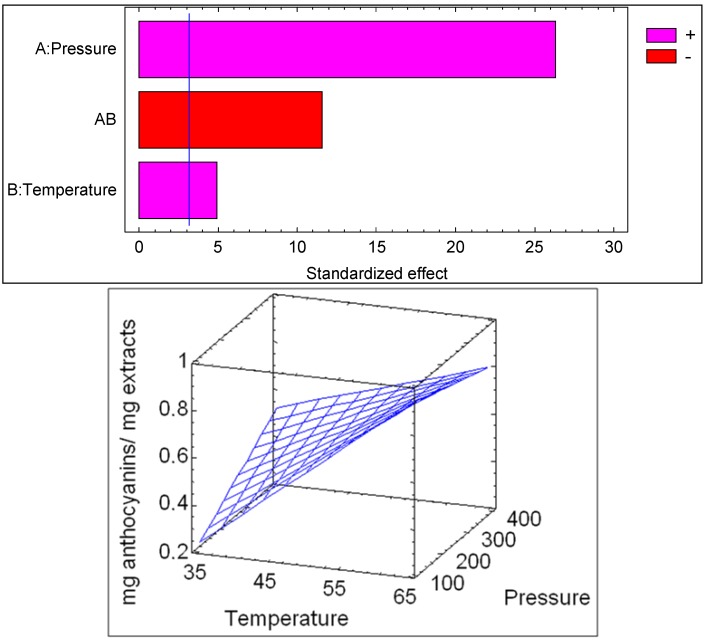
Pareto diagram and response surface for the SFE of anthocyanins with respect to extraction temperatures and pressures.

The relationship between anthocyanins in the extract and the main variables is depicted in Figure 3. The Pareto diagram of significance of each factor is represented; when a factor has a bar greater than the blue line it influences the process in a significant way for a confidence level of 0.05. The sign associated with each of the effects indicates a positive or negative influence on the yield caused by the increase of the variable.

The relationship between temperature and pressure for the extraction of anthocyanin compounds is represented with a good coefficient (R^2^ = 0.9964) by Equation (1):

Y = 0.6237 + 0.4725P + 0.0875T − 0.2075PT
(1)

where Y represents mg anthocyanins/mg extract; P the pressure (bar) and T the temperature (°C).

### 2.2. Pressurized Liquid Extraction (PLE)

On the basis of the results obtained in the SFE study, the highest anthocyanin contents were obtained under conditions that gave the lowest extraction yields (100 bar, 65 °C). Furthermore, bearing in mind that the material extracted in this process retained the anthocyanin color, it was decided to study an alternative extraction technique. In this sense, pressurized liquid extraction (PLE) is becoming increasingly important as a sample preparation technique in food analysis as it combines the benefits of high-throughput, automation and favorable environmental impact due to the low solvent consumption [20]. PLE enables the rapid extraction of analytes in a closed and inert environment under high pressures and temperatures. A major advantage of PLE over conventional solvent extraction methods conducted at atmospheric pressure is that pressurized solvents remain in the liquid state well above their boiling points, thus allowing high-temperature extractions. These conditions improve analyte solubilities and the desorption kinetics from the matrices.

**Figure 4:**
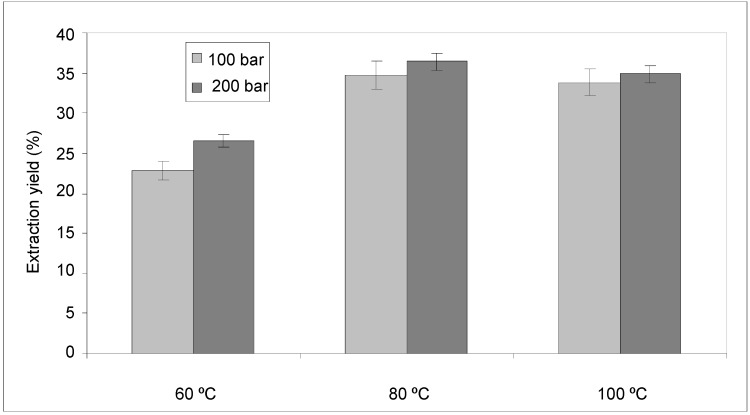
Extraction yield of extracts obtained by PLE at different pressures and temperatures.

One disadvantage of this technique is that the extraction conditions (solvent, pressure, temperature) must be studied for each material given the discrepancies in the results reported in the literature. The use of this technique for the extraction of anthocyanins from potato peels has not been reported in the literature, however, several authors have developed methods for the PLE extraction of anthocyanins from other materials using acidified water and acidified methanol or ethanol as solvents [21,22]. The solvent used in this work has been: 80% (v/v) of ethanol in water acidified to pH 2.6 with acetic acid [23]. The influence of pressure and temperature on the extraction yield was investigated. Figure 4 shows the data obtained, it was found to increase significantly in comparison to the results obtained with SFE.

Temperature is a variable that affects the extraction efficiency of PLE. Raising the temperature increases the diffusion rates, the solubility of the analytes and the mass transfer but it decreases the viscosity and surface tension of the solvents. These changes improve the contact of the analytes with the solvent and enhance the extraction, which can then be achieved more rapidly and with lower solvent consumption compared with classical methods. In the PLE procedure, fast and efficient extraction is achieved by applying elevated temperatures from 60 °C to 80 °C.

Among the variables that affect the performance of the PLE procedure, pressure is another important parameter and extraction efficiencies generally increase with elevated pressure. High pressure helps to force the solvent into the sample matrix pores and to keep the solvent in the liquid state at the operating temperature. In the work described here, the best extraction yields were obtained at 200 bar.

#### 2.2.1. Anthocyanin and Phenolic Contents

**Figure 5:**
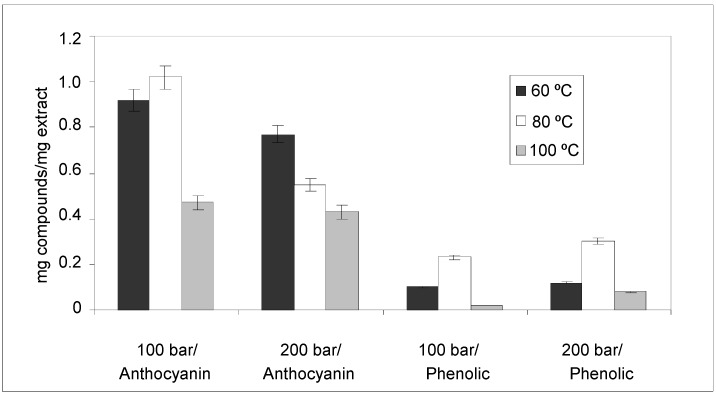
Anthocyanin and phenolic concentration of extracts obtained by PLE at different pressures and temperatures.

The anthocyanin concentration (mg anthocyanins/mg dry extract) and phenolic total contents (mg phenolic total/mg dry extract) in the extracts obtained by PLE is show in Figure 5. At 100 bar a raising the temperature for 60 °C to 80 °C increases the anthocyanin and phenolic compound content due an increases the diffusion rates, the solubility of the analytes and the mass transfer. The decrease in anthocyanin and phenolic contents at high temperatures could be due to degradation caused by hydrolysis. Increasing the extraction temperature also led to a turbid or cloudy appearance in the extracts, whereas the extract was clear at 80 °C.

The effect of pressure in PLE is not always the same. Studies performed by Luthria [24] and Mukhopadhyay *et al.* [25] indicated that pressure does not have a significant effect on the extraction of phenolic compounds from natural products. In this study, increasing the pressure from 100 bar to 200 bar, increased the phenolic compound content. The results obtained are in accordance with those of Singh *et al.* [14], who studied extraction and stability of phenolics extracted from waste potato peel using MAE.

By the other hand, when the extraction of anthocyanins is analyzed, the effect observed is different. The best yields were obtained at 100 bar and 80 °C. This result could be due to the fact that higher pressure (200 bar) favors the coextraction of other analytes such as phenolic compounds. 

**Figure 6:**
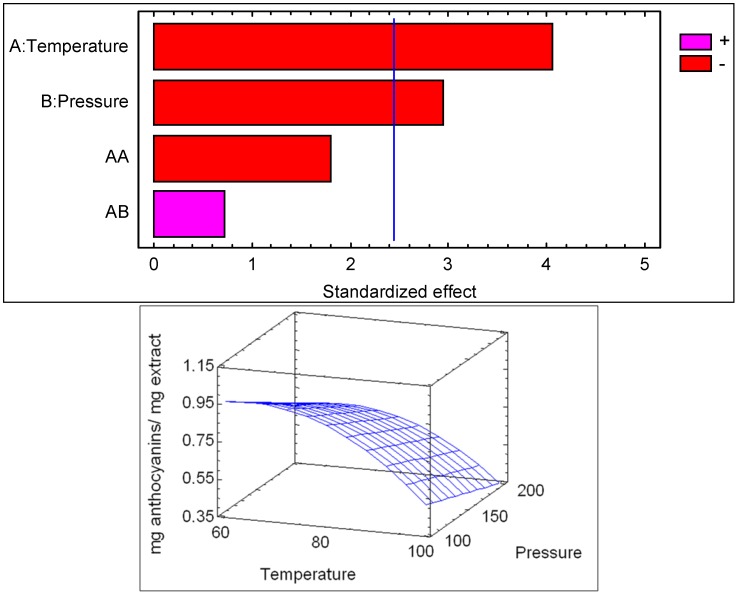
Pareto diagram and response surface for the PLE of anthocyanins with respect to extraction temperatures and pressures.

Due to the fact that the main compounds presented in the extract are anthocyanins, the content of these compounds obtained by PLE was statistically analyzed. The ANOVA results (Table 2) show that the extraction temperature was highly significant (*p* < 0.05) in the extraction of anthocyanin compounds. The effect of pressure was also significant (*p* < 0.05), while the crossed pressure-temperature interaction and quadratic temperature interaction were insignificant. The relationship between anthocyanins in the extract and the main variables is depicted in Figure 6. The Pareto diagram of significance of each factor is represented; when a factor has a bar greater than the blue line it influences the process in a significant way for a confidence level of 0.05. The sign associated with each of the effects indicates a positive or negative influence on the yield caused by the variation of the variable.

**Table 2:** ANOVA for the effect of pressure (P) and temperature (T) on anthocyanin contents.

Source	SS	DF	MS	F-Value	*p*-value
P	0.154133	1	0.154133	8,61	0.0261
T	0.292613	1	0.292613	16,35	0.0068
PT	0.0091125	1	0.0091125	0,51	0.5023
T^2^	0.0570375	1	0.0570375	3.19	0.1245
Blok	0.0003	1	0.0003	0.02	0.9012
Total error	0.107404	6	0.0179007		
Total (corr.)	0.6206	11			

The relationship between temperature and pressure for the extraction of anthocyanin compounds is represented with a good coefficient (R^2^ = 0.988) by Equation (2):

Y = 0.7675 − 0.3825T − 0.2266P − 0.2925T^2^ + 0.0675PT (2)

where Y represents mg anthocyanins/mg extracts; P the pressure (bar) and T the temperature (°C).

### 2.3. Antiradical Activities of the Extracts

The antiradical activities of the extracts obtained were assessed using the 2,2-diphenyl-1-picrylhydrazyl (DPPH) radical scavenging assay. This technique was only applied to the extracts obtained by PLE because this extraction gave greater quantities of anthocyanins. The DPPH assay is a quick, reliable and reproducible method to assess the antiradical activities of pure compounds as well as plant extracts. This method depends on the reduction of the purple DPPH to give a yellow colored diphenyl picrylhydrazine and measurement of the remaining DPPH. According to the classification developed by Scherer and Godoy [2] and the results shown in Table 3, the extracts obtained at 60 °C and 100 °C show moderate antioxidant activity because the antioxidant activity index (AAI) values are between 0.5 and 1.0 approximately. However, for the extract obtained at 80 °C the AAI values were between 1.0 and 2.0, which indicates that these extracts show strong antioxidant activity.

**Table 3:** Antioxidant activity expressed as AAI of extracts obtained by PLE under different extraction conditions.

Pressure	60 °C	80 °C	100 °C
100 bar	0.91	1.66	0.72
200 bar	1.05	1.53	0.80

The results of 80 °C, 200 bar in Figure 5 show that a lesser amount of anthocyanins was extracted than at 60 °C, 200 bars, but the extract had higher AAI than that at 60 °C, 200 bar did. This behavior can be attributed to the fact that anthocyanins are not the only compounds responsible of the antioxidant capacity of the obtained extracts. The concentration of phenolic compounds in the extract can modify this property. At 80 °C and 200 bar, the results obtained show that the phenolic compounds are in a greater concentration than 60 °C and 200 bar. The higher concentration of these compounds can compensate the decrease in the concentration of anthocyanins in the extract.

The results are similar to those reported by Ghafoor *et al.* [22] for anthocyanins in extracts from grape (*Vitis labrusca* B.) peel. The minimum value obtained for these was 0.52 and the maximum was 1.24. Bearing in mind the results for antioxidant capacity and the yields of anthocyanins in the extracts, the conditions 100 bar and 80 °C appear to be the best for the extraction of anthocyanins from *Solanum stenotomun* using the PLE technique.

## 3. Experimental

### 3.1. Samples and Chemicals

The raw material used was obtained from Bolivian potatoes (*Solamun stenotomun*) of the variety ‘Pinta-Boca’. Samples were liophilized to constant weight. All results are expressed on dry basis. Carbon dioxide (99.995%) was supplied by Abello-Linde S.A. (Barcelona, Spain). 2,2-Diphenyl-1-picrylhydrazyl, free radical (DPPH), pelargonin chloride, malvin chloride, cyanin chloride and gallic acid standards were supplied by Sigma-Aldrich (Steinheim, Germany). The organic solvents ethanol, methanol and acetic acid, all HPLC gradient grade, were supplied by Panreac (Barcelona, Spain). The water used in all experiments was double-distilled MilliQ grade.

### 3.2. Extractions with Solvents at High Pressures

Extraction tests were carried out in a model SF100 high-pressure apparatus supplied by Thar Technology (Pittsburgh, PA, USA). This set-up included an extraction vessel (100 mL capacity) with a thermostatic jacket to control the extraction temperature, two pumps with a maximum flow rate of 50 g/min (one for carbon dioxide and the other for cosolvent), a back pressure valve regulator to control the system pressure, and a cyclonic separator to allow periodic discharge of the extracted material during the extraction process. For all tests the extraction vessel was loaded with approximately 30 g of sample. Extracts were recovered in a cyclonic separator and then collected in amber glass bottles.

The effects of different variables on the SFE were analyzed by considering the following operating conditions: pressures of 100 and 400 bar, temperatures of 35 and 65 °C, cosolvent percentages of 0 and 5% (v/v). All tests were carried out with a carbon dioxide flow rate of 20 g/min and an extraction time of 3 h. Ethanol was employed as a cosolvent because subcritical fluid extraction with a high percentage of cosolvent is too efficient for the extraction of anthocyanins [26]. In addition, this solvent is considered to be a green solvent whose use in the pharmaceutical and food industries is not restricted. Extracts were dried under vacuum at 35 °C, weighed in order to determine global extraction yields and then stored in darkness at −20 °C prior to assays. 

The effects of the same variables (pressure and temperature) on the PLE were analyzed. In this case the temperature was set at 60, 80 and 100 °C and the pressures used were 100 and 200 bar. The composition of solvent was 80% (v/v) of ethanol in water; this mixture was acidified to pH 2.6 with acetic acid [23]. The acidification of the solvent was carried out by adding the organic acids to the solvent while measuring the pH using a Mettler Toledo (Columbus, OH, USA) SevenEasy S-20 pH meter fitted with a pH electrode. All tests were carried out with a flow rate of 10 g/min and an extraction time of 3 h. Extracts were stored in darkness at −20 °C prior to assays. 

### 3.3. Anthocyanins Analysis

HPLC analysis of the anthocyanins present in the extracts was performed using the method described by Cho *et al.* [27]. An Agilent Technologies (Palo Alto, CA, USA) 1100 Series chromatograph was used. 5% Formic acid in water (v/v) (A) and methanol (B) were used as solvents in this HPLC method. The HPLC gradient program was executed as follows: 98% A to 40% A in 60 min, 40% A to 98% A in 5 min. The entire HPLC run time was 70 min using a flow rate of 1 mL/min. 

The resultant extracts were filtered before HPLC assay using a 0.45 μm PTFE filter (Varian Inc., Palo Alto, CA, USA) and 100 μL of the filtered extract was injected into the column (250 × 4.6 mm) C18 Hypersil ODS (5 μm particle size) (Supelco). The compounds were detected using a UV-Vis detector at a wavelength of 510 nm. The peaks were identified by comparison of retention times with those of the commercial standards (Sigma) and were quantified by means of calibration curves. The anthocyanins in the extract analyzed by HPLC were reported based on malvin chloride. The experiments for each extraction were carried out in triplicate in order to evaluate the variability of the measurements. The results are shown as the average of all the independent analyses with a reproducibility of approximately 4.5% CV (coefficient of variation).

### 3.4. Phenolic Compounds Analysis

Shimadzu (Tokyo, Japan) UVmini-1240 spectrophotometer was used for analytical determination of phenolic compounds. A mixture of 0.2 mL of each extract and 3.8 mL of hydrochloric acid (1 M) was introduced in the flask [28]. After 1 h the solutions were measured at 280 nm. Gallic acid was used as standard. The experiments for each extraction were carried out in triplicate in order to evaluate the variability of the measurements. The results are shown as the average of all the independent analyses with a reproducibility of approximately 3.2% CV (coefficient of variation).

### 3.5. Antioxidant Assay with DPPH

The antioxidant activities were determined using DPPH as a free radical. Different concentrations were tested (expressed as the mg of extract/mg DPPH) for each set of extraction conditions. Extract solution in methanol (0.1 mL) was added to a 6 × 10^−5^ mol/L methanol DPPH solution (3.9 mL). The decrease in absorbance was determined at 515 nm at different times until the reaction had ‘reached a plateau’. The exact initial DPPH concentration (CDPPH) in the reaction medium was calculated from a calibration curve with the following equation:

Abs_515nm_ = −0.0065 × 29.3112(CDPPH) (3)

as determined by linear regression (r = 0.9999).

For each set of extraction conditions a plot of % remaining DPPH *vs.* time (min) was generated. These graphs were used to determine the percentage of DPPH remaining at the steady state and the values were transferred onto another graph showing the percentage of residual DPPH at the steady state as a function of the weight ratio of antioxidant to DPPH. Antiradical activity was defined as the amount of antioxidant required to decrease the initial DPPH concentration by 50% [Efficient Concentration = EC_50_ (mg extract/mg DPPH)]. The antioxidant activity was expressed as the antioxidant activity index (AAI), which was calculated in terms of 1/EC_50_. 

The experiments were carried out in triplicate in order to evaluate the variability of the measurements. The results are shown as the average of all the independent analyses and the reproducibility was approximately 1.9% CV (coefficient of variation).

### 3.6. Experimental Design

Given the importance of obtaining extracts with high anthocyanin contents, experimental designs were carried out for the extracts obtained by SFE and PLE. Due to the fact that the objective of the paper was to analyze the influence of operating conditions in the anthocyanins yield, a screening experimental design was selected. The variables studied were pressure and temperature. In the case of SFE, a 2^2^ factorial design was carried out, whereas for PLE a multifactorial design was carried out. The factor and physical values are shown in Table 4. The experiments were performed in randomized order.

**Table 4:** Physical values in the experimental design.

Extraction method	Experimental variable	Factor	Levels			
SFE	Pressure	P	100 bar		400 bar	
	Temperatue	T	35 °C		65 °C	
PLE	Pressure	P	100 bar		200 bar	
	Temperatue	T	60 °C	80°C		100 °C

Statistical calculations and analyses were performed using STATGRAPHICS Plus 5.1® (1994–2001, Statistical Graphics Corp.). An empirical correlation was developed in order to predict the influence of extraction conditions on the extraction yields of anthocyanins.

## 4. Conclusions

The results presented in this study show PLE is an excellent alternative for the recovery of high quantities of anthocyanins from *Solanum stenotomun* peels, and more efficient than supercritical CO_2_ + ethanol. SFE with pure CO_2_ is not an adequate technique for the extraction of anthocyanins, but the addition of 5% (v/v) ethanol cosolvent to the CO_2_ improves the extraction yield and anthocyanin contents. In SFE with CO_2_ + ethanol the use of low pressure (100 bar) and high temperature (65 °C) was desirable for the extraction of anthocyanins. In PLE the best yield of anthocyanins were obtained at 100 bar and 80 °C. PLE extracts obtained at 80 °C presented a potent antioxidant activity.

The results obtained make the high-pressure technology applied to extraction process a promising process for scale up. Nevertheless, in order to determine the capability of an industrial process, pilot plant tests at higher scale will be necessary. Comparing the results obtained at both scales, and applying the similarity criteria, it will be possible to analyze the economic viability of the process.
